# Global Oral Health Policies and Guidelines: Using Silver Diamine Fluoride for Caries Control

**DOI:** 10.3389/froh.2021.685557

**Published:** 2021-07-30

**Authors:** Sherry Shiqian Gao, Gwendolyn Amarquaye, Peter Arrow, Kalpana Bansal, Raman Bedi, Guglielmo Campus, Kitty Jieyi Chen, Ana Cláudia Rodrigues Chibinski, Tselmuun Chinzorig, Yasmi O. Crystal, Duangporn Duangthip, María Laura Ferri, Morenike Oluwatoyin Folayan, Ariuntuul Garidkhuu, Hamdi H. Hamama, Varangkanar Jirarattanasopha, Arthur Kemoli, Soraya C. Leal, Pattarawadee Leelataweewud, Vijay Prakash Mathur, Tshepiso Mfolo, Yasuko Momoi, Nicoline Potgieter, Arzu Tezvergil-Mutluay, Edward Chin Man Lo, Chun Hung Chu

**Affiliations:** ^1^Faculty of Dentistry, The University of Hong Kong, Hong Kong, China; ^2^Dental Unit, Korle-Bu Teaching Hospital, Accra, Ghana; ^3^Department of Orthodontics and Pedodontics, University of Ghana Dental School, Ghana College of Physicians and Surgeons, University of Ghana, Accra, Ghana; ^4^WA Dental Health Services, Perth, WA, Australia; ^5^Dental School, The University of Western Australia, Perth, WA, Australia; ^6^Dental School, The University of Adelaide, Adelaide, SA, Australia; ^7^Department of Pediatric & Preventive Dentistry, Centre for Dental Education & Research, All India Institute of Medical Sciences, New Delhi, India; ^8^King's College London, London, United Kingdom; ^9^C/o Global Child Dental Fund, London, United Kingdom; ^10^Department of Restorative, Preventive and Pediatric Dentistry, University of Bern, Bern, Switzerland; ^11^Department of Surgery, Microsurgery and Medicine Sciences, School of Dentistry, University of Sassari, Sassari, Italy; ^12^Guanghua School of Stomatology, Hospital of Stomatology, Sun Yat-sen University, Guangzhou, China; ^13^Faculty of Dentistry, State University of Ponta Grossa, Ponta Grossa, Brazil; ^14^Graduate School of Mongolian National University of Medical Sciences, Ulaanbaatar, Mongolia; ^15^Division of Research and Treatment for Oral and Maxillofacial Congenital Anomalies, School of Dentistry, Aichi-Gakuin University, Nagoya, Japan; ^16^Pediatric Dentistry Department, New York University School of Dentistry, New York, NY, United States; ^17^Pediatric Dentistry Postgraduate Department, School of Dentistry, Maimonides University, Buenos Aires, Argentina; ^18^Pediatric Dentistry Department, School of Dentistry, University of El Salvador, San Salvador, Argentina; ^19^Faculty of Dentistry, Obafemi Awolowo University, Ife, Nigeria; ^20^School of Dentistry, Mongolian National University of Medical Sciences, Ulaanbaatar, Mongolia; ^21^Department of Public Health, School of Medicine, International University of Health and Welfare, Narita Campus, Japan; ^22^Operative Dentistry Department, Faculty of Dentistry, Mansoura University, Mansoura, Egypt; ^23^Department of Pediatric Dentistry, Faculty of Dentistry, Mahidol University, Bangkok, Thailand; ^24^School of Dental Sciences, University of Nairobi, Nairobi, Kenya; ^25^Departamento of Dentistry, Faculty of Health Sciences, University of Brasília, Brasília, Brazil; ^26^Department of Community Dentistry, University of Pretoria, Pretoria, South Africa; ^27^School of Dental Medicine, Tsurumi University, Yokohama, Japan; ^28^Department of Paediatric Dentistry, University of the Western Cape, Cape Town, South Africa; ^29^Department of Cariology and Restorative Dentistry and Cariology, Turku University Hospital, University of Turku, Turku, Finland

**Keywords:** silver diamine fluoride, fluoride, dental caries, early childhood caries, root caries, COVID-19

## Abstract

Silver diamine fluoride (SDF) was developed in Japan in the 1960s. It is a clear solution containing silver and fluoride ions. Because of its anti-bacterial and remineralizing effect, silver diamine fluoride has been used in managing dental caries for decades worldwide. This paper aims to summarize and discuss the global policies, guidelines, and relevant information on utilizing SDF for caries management. SDF can be used for treating dental caries in most countries. However, it is not permitted to be used in mainland China. Several manufacturers, mainly in Australia, Brazil, India, Japan, and the United States, produce SDF at different concentrations that are commercially available around the world. The prices differ between contents and brands. Different government organizations and dental associations have developed guidelines for clinical use of SDF. Dental professionals can refer to the specific guidelines in their own countries or territories. Training for using SDF is part of undergraduate and/or postgraduate curriculums in almost all countries. However, real utilization of SDF of dentists, especially in the private sector, remains unclear in most places because little research has been conducted. There are at least two ongoing regional-wide large-scale oral health programs, using SDF as one of the components to manage dental caries in young children (one in Hong Kong and one in Mongolia). Because SDF treatment does not require caries removal, and it is simple, non-invasive, and inexpensive, SDF is a valuable strategy for caries management in young children, elderly people, and patients with special needs. In addition, to reduce the risk of bacteria or virus transmission in dental settings, using SDF as a non-aerosol producing procedure should be emphasized under the COVID-19 outbreak.

## Introduction

Silver diamine fluoride (SDF) was developed in the 1960s by Professor Reiichi Yamaga from Osaka University, Japan [1]. It is a clear ammonia solution, containing silver, and fluoride ions. SDF was first introduced to arrest dental caries in children. The arresting technique was based on the disinfectant character of silver and the remineralizing effect of fluoride. During the era of high caries prevalence in children and the shortage of dentists in Japan (around the 1970s), SDF was widely adopted in community dental clinics to manage dental caries in young children [1]. However, its use has become uncommon in Japan since the 1990s when the restorative materials and techniques had been well-developed.

Nevertheless, in recent years, SDF has gained popularity in dental research and dental clinical work in the rest of the world. Laboratory studies, using advanced technology proved that SDF has an anti-bacterial effect, can enhance the remineralization of dentine tissue and can protect the dentine collagens [2]. Several well-designed clinical studies supported that SDF was effective in arresting dentine caries [3]. Systematic reviews have reported that 38% SDF is effective in arresting early childhood caries (ECC); around 80% of the carious surface became arrested after SDF application [3]. SDF also shows a good preventive and arresting effect on carious root surfaces [4]. Apart from the black staining of the carious lesion, no other severe adverse effect has been reported [5]. Because of its relatively low cost and simplicity of application, SDF was considered a non-invasive and promising strategy to manage dental caries, especially when the conventional restorative treatments were not applicable, available, or affordable in some cases.

Through a literature search, we found that most of the clinical research on SDF was conducted in some particular countries, including Brazil, China, Japan, and the United States (US). Some countries may have published SDF-related articles; however, these articles were in their own languages that could not be searched, using English keywords. Therefore, it was difficult to find information on SDF use in other parts of the world. Moreover, we found that the protocols or recommendations of using SDF in managing dental caries suggested in different studies or articles varied significantly. There is no gathered information of the policies and guidelines of SDF use in different countries in the literature. Therefore, this paper aims to summarize and discuss the global policies, guidelines, and relevant information of using SDF in managing dental caries worldwide.

## Argentina

The use of SDF in dentistry is not a novelty in Argentina. It has been used since 1978, both in public health and private practice. Until now, the Argentine Health Ministry had not established policies and guidelines for using SDF. However, creating a committee of experts in oral health is in progress. The committee will develop standardized policies and provide preventive guidelines to reduce high dental caries prevalence, and the use of SDF will be included [6]. In addition, there are no uniform guidelines for using SDF in the different organizations in Argentina; each institution has its own recommendations for clinical applications of SDF. According to the present finding, in different Argentina provinces, we were informed that there are no documented data on using SDF in private practice. Most health insurance systems do not cover a specific dental treatment with SDF, but some include it as an application of remineralizing substances. The majority of public and private universities include the use of SDF in their curriculums in both undergraduate and postgraduate programs, especially in Pediatric Dentistry, Cariology, Dental Materials and Preventive Dentistry departments. Each university implements its own clinical protocols and recommendations for using SDF. In addition, SDF has been used for several decades for managing dental caries in community oral health programs in highly vulnerable populations with limited access to dental care.

Currently, there are two local companies developing an SDF product. One of these is Tedequim whose trade name is Fagamin. Its product consists of 38% SDF (pH 7–9); the presentation is a 5-ml bottle, approved by National Administration of Drugs, Food and Medical Devices (ANMAT) in 1991 [7]. The other company is Densell whose brand name is Fluorsilver. Its product also consists of 38% SDF solution presented in a 5-ml bottle and was approved by ANMAT in 2009 [8]. There are also imported products commercially available in the Argentinian market, which are Cariestop (Biodinamica, Brazil) and Riva Star (SDI Limited, Australia).

## Australia

Australia has a history of using silver fluoride (SF), which commenced with the study of Craig et al. in the early 1980s to limit caries progression in primary teeth [9]. The West Australian School of Dental Service adopted the protocol service wide in the early 1980s. Recent research has examined the application of SF as a caries treatment agent in remote Indigenous communities and found it to be as effective as atraumatic restorative treatment (ART) [10]. Also, the most recent Australian consensus guideline on the appropriate use of fluorides in Australia stated that using SF or SDF might be indicated where “traditional treatment approaches to caries management might not be possible due to behavioral or medical management challenges, or where access to care is difficult or not available [11].”

There are no uniform guidelines for using SDF within oral health schemes in Australia; public dental services of each state and territory have their own policy and guidelines on dental treatment procedures. SDF is not used at all in some jurisdiction, while, in others, its use is incorporated into the clinical service manuals for the treatment of carious primary and permanent teeth. Currently, the Australian Therapeutics Goods Administration has not approved it as a therapeutic agent for treating dental caries; however, it is used off labelly in caries management in general practices and public dental services [12]. The application of the agent may be classified under the dental fee schedule for applying a remineralizing agent or desensitizing procedures, but there is no specific billing or reimbursement for the application of SDF. In addition, there does not appear to be specific training of SDF at the undergraduate or postgraduate level within the dental faculties in Australia.

Currently, SDF is available for treatment as a two-part tooth desensitizing agent (Riva Star, SDI Limited, Australia) with an SDF component (comprising SF 35–40% by weight; ammonia 15–20% by weight and the remaining component, water) and the potassium iodide (KI) component to reduce staining. It is sold as a kit in individual-use, prepacked capsules (10 capsules of 0.05-ml SDF and 10 capsules of KI), or in a two-bottle kit (1.5-ml SDF and 3.-ml KI). SF is also available and sold as an agent for disclosing the caries status of a tooth [marketed as caries status disclosing solution (CSDS)] in a two-bottle kit (3 ml, 40% SF solution and 3 ml, 10% stannous fluoride solution) (RM Creighton Dental Pty Ltd., Australia).

## Brazil

Professor Tadaaki Ando from University of São Paulo brought SDF to Brazil after one trip to Japan in the 1980s. He developed a Brazilian product, using the original compounds from the Japanese solution (silver nitrate, hydrofluoric acid, sodium hydroxide, and ammonia) and called it “cariostático” (cariostatic solution). Around the same time, Professor Luiz Walter from Londrina State University developed a 30% SDF solution, named it “Safluoride de Walter” and started to recommend its use in clinics of children for treating active dentine carious lesions. Since then, SDF use has become popular in Brazil in different concentrations (10, 12, and 30%). Three local brands of SDF are commercially available in the Brazilian market, which are Cariestop by Biodinâmica, Cariostasul by Iodontosul, and Ancárie by Maquira. Riva Star is also imported and available in Brazil (Table 1). In 2020, the Brazilian Ministry of Health (BMH) launched a collaborative document with the participation of different health councils, including the Brazilian Association of Dentistry, to describe the protocols in primary health care for the Brazilian public health system [13]. The use of SDF is listed under the section “Attention and Care Related to Oral Health,” described as a procedure that aims to “control the demineralization of the dental surfaces.” The document recommends using a 30% SDF solution but does not mention the kind of patient or the clinical situations in which SDF should be used. The Brazilian Association of Pediatric Dentistry has recently published the “Guidelines for Clinical Procedures in Pediatric Dentistry” [14], in which using SDF is indicated for children with multiple active dentin carious lesions, children with behavioral problems that are not cooperative with the traditional treatments, early childhood caries, cavitated carious lesions that are difficult to restore, and for patients who have restricted access to dental services. Using SDF to control enamel carious lesions in pit and fissures or in proximal enamel lesions is also cited, with the observation that the quality of the evidence of those topics is weaker than the evidence of its use for controlling dentin carious lesions. Moreover, the document highlights that the SDF treatment should be provided in association with the control of the etiological factors related to the caries disease. The schemes and reimbursement related to using SDF vary according to system in consideration. Although BMH recommends SDF, the cost for individual application is not available, as the reimbursement system does not consider specific procedures but allocates funds according to the size of the population and the number of oral health teams in a given municipality. A considerable number of Brazilian universities include SDF in the dental curriculum; the subject is taught mainly in pediatric dentistry and cariology courses as part of the minimally invasive strategies used to treat dental caries.

**Table 1:** Global policies, guidelines, and relevant information of SDF for caries management.

**Country**	**Availability**	Brand (local): concentration of SDF/content/price^*^	**Guidelines**	**Policies of SDF use in healthcare system**	**National/regional healthcare programme**	**Dental training**
Argentina	Yes	Fagamin: 38%/5 mL/20 Fluorsilver: 38%/5 mL/15 Imported products: Riva Star, Cariestop	Each oral health organization has its own recommendations	Different recommendations in different regions	Regional programmes	For both undergraduate and postgraduate
Australia	Yes	Riva Star^a^:35–40%/0.5 mL (10 capsules)/89.835–40%/1.5 mL (bottle)/89.8 CSDS^b^: 40%/3 mL/69.2	Australian fluoride guidelines [11]	*ad hoc* by states and territories	*ad hoc* by states and territories	No specific training
Brazil	Yes	Cariestop:12%/10 mL/8.4 30%/5 mL/8.4 Cariostasul: 10%/10 mL/3.2 12%/10 mL/3.6 Ancárie: 12%/10 mL/3.7 30%/5 mL/5.2 Imported products: Riva Star	National Protocol in Primary Health Care for the Brazilian public health system [13] Guidelines for Clinical Procedures in Pediatric Dentistry [14]	Different policies in different localities around the country	No	For both undergraduate and postgraduate
China (Mainland)	No	N/A	N/A	N/A	N/A	Only included in the pediatric textbook for undergraduates
China (Hong Kong)	Yes	Imported products: Advantage Arrest, Cariestop, Riva Star, Saforide	No	No	Yes, a territory-wide service programme [15]	For both undergraduate and postgraduate
Egypt	Yes	Imported products: e-SDF, Riva Star	No	No	No	Most in clinical research in universities
Finland	Yes	Imported products: Riva star	National guidelines for caries management [16]	Reflected in the recommendations	No	For both undergraduate and postgraduate
Ghana	Yes	Imported products: e-SDF	No	No	No	For postgraduate training only
India	Yes	e-SDF:38%/5 mL/34 Imported products: Cariestop, Dengen Caries Arrest, Fagamin	No	No	No	Undergraduate (rare) Postgraduate (yes)
Japan	Yes	Saforide:38%/5 mL/60.3	Clinical Practice Guideline for Caries Treatment [17]	Included in national health insurance	No	For both undergraduate and postgraduate
Kenya	Yes	Imported products: Advantage Arrest, e-SDF, Fagamin	No	No	No	For both undergraduate and postgraduate
Mongolia	Yes	Imported products: Saforide	Standard guideline and recommendation of SDF	Reflected in the Standard Guideline and Recommendation of SDF	“Healthy teeth-healthy child” National Program (2019-2023)	Included in the undergraduate curriculum and the National Examination of dental license
Nigeria	Yes	Imported products: Advantage arrest	No	No	*ad hoc* outreach and clinical programs	Only one out of eleven dental schools include SDF in undergraduate and postgraduate training
South Africa	Yes	Imported products: e-SDF, Riva star	No	No	No	For both undergraduate and postgraduate
Switzerland	Yes	Imported products: Riva star	Clinical guidance and recommendation regarding the management of caries [18–21]	Reflected in the recommendations	No	For both undergraduate and postgraduate
Thailand	Yes	Topamine^c^:38%/5 mL/88	Guideline on caries diagnosis and management [22]	Covered by universal health coverage	No	For both undergraduate and postgraduate
The United Kingdom	Yes	Imported products: Riva Star	Standard operating procedure (England) [23] British Society of Pediatric DentistryNew SDF resources [24]	Reflected in the recommendations	No	For both undergraduate and postgraduate
The United States	Yes	Advantage arrest:38% SDF/8 mL/168	American academy of pediatric dentistry guideline 2017 [25] Evidence-based clinical practice guideline on nonrestorative treatments for carious lesions [26]	Policy on the use of silver diamine fluoride for pediatric dental patients [27]	*ad hoc* by States	Being taught didactically and used in the clinic in 100% of pediatric dental programs in the United States.

*SDF, silver diamine fluoride; ml, millimeter; CSDS, caries status disclosing solution; Ref, reference*.

* *Price in the United States dollars*.

a *Riva Star includes SDF component and potassium iodide component*.

b *CSDS is a two-bottle kit that includes 40% silver fluoride solution and 10% stannous fluoride solution*.

c *Topamine is produced by PharmaDesign Co., Ltd. (a division of Dentalife Australia Pty Ltd) in Thailand*.

According to the official database that takes into account the public oral health system, in the last 5 years (2015 to 2020), more than 3,775,000 SDF applications (a tooth level) were performed in the country. However, a decrease in use was observed along the years. In 2015, there were 1,176,710 applications performed, followed by 990,980 (2016); 813,592 (2017); 396,058 (2018); 309,826 (2019) to <88,000 in 2020. Studies have reported that dentists tend to exhibit some prejudice regarding SDF treatment, which hampers the use of the product [28, 29]. On the other hand, there are studies showing that the acceptance of the patients of SDF was considerably high [30, 31]. Parents were more interested in the positive treatment results than in aesthetics [31]. Regardless of positive perceptions of patients, note that the aforementioned research was carried out in public schools or in public health centers, and these results may not be representative of the entire Brazilian population.

## China (Mainland)

Although Chinese researchers have conducted several clinical studies to investigate the effectiveness of SDF in managing dental caries and favorable results were reported [32–34], SDF has not obtained approval from the Chinese National Medical Products Administration [35]. It is not permitted to be used on patients in dental hospitals or clinics. The Chinese textbook *Pediatric Dentistry* for undergraduate dental students still includes one short chapter on SDF [36]. It is introduced as a non-invasive strategy to treat dental caries on primary dentition. However, in the undergraduate training, conventional restorative treatments are considered more favorable than SDF in managing dental caries.

## China (Hong Kong)

Because of the “one country, two systems” principle, Hong Kong has a different healthcare system from mainland China, and SDF is permitted to be used in Hong Kong. The University of Hong Kong (HKU) has been extensively studying SDF in the past 20 years. More than 70 articles on its use for caries management were published. In response to the high caries prevalence of ECC and need of residents for affordable and accessible dental treatment, HKU established a pilot preschool dental care service, using SDF in 2008. Later, the project expanded to serve ~100 kindergartens in 2010–2019. The headmistresses, teachers, and administrative staff from kindergartens collaborated with HKU and the children and their parents highly appreciated the service. No significant complications after SDF treatment were reported [5]. Several clinical trials on SDF conducted in Hong Kong children confirmed the effectiveness of SDF for caries arrest in primary teeth [37, 38]. For the aged population, effectiveness of SDF in preventing and arresting root caries was also evaluated, and the findings were published [39]. Recently, the Faculty of Dentistry of HKU has launched a new project named Jockey Club Children Oral Health Project [15]. This large-scale, territory-wide project, supported by the Hong Kong Jockey Club Charities Trust, aims to provide dental screening, uses SDF to control ECC in preschool children, and enhances dental knowledge and attitudes of parents and teachers. At present, all Hong Kong kindergartens are being invited to join this free outreach dental service. Results of project activities and lessons learned will be reported in the future.

Currently, only a few SDF brands are commercially available in Hong Kong (Table 1). No information exists about to what extent SDF has been used as an alternative for caries control in the private sector. There is a lack of information on perspectives and experiences of dental professionals of using SDF in Hong Kong. In dental education, SDF is now taught in three main disciplines: cariology, pediatric dentistry, and dental public health. So far, the Department of Health or Dental Societies has not published official guidelines of SDF treatment in Hong Kong.

## Egypt

Although studies have reported the effectiveness of SDF in treating dental caries, general dentists in Egypt have recently become aware of this agent from university staff (particularly from Operative Dentistry and Dental Public Health Departments), and the overall use of SDF in the private sector is still questionable. Many dentists in this region only know SDF by name; however, they are not willing to try it on their patients. This might be attributed to the concerns over teeth discoloration, following SDF application.

The available SDF brands in Egypt are Riva Star (SDI Limited, Australia) and e-SDF (Kid-E-Dental, India). e-SDF consists of a 38% SDF solution, and pediatric dentists commonly use it. The distributor of Riva Star reported to us that most of its clients are academicians or postgraduate students who conducted research projects in governmental universities in Cairo, Mansoura, Ain-Shams, and Alexandria. The distributor also stated that awareness of general dentists about Riva Star needs improvement. Conversely, the e-SDF agent distributor stated that it has many clients working in general dentistry, and they mainly use this product for children.

Since 2015, the Operative Dentistry Department of Mansoura University has implemented teaching SDF as one of the possible treatments of incipient carious lesions in the operative dentistry curriculum in the fourth year of undergraduate training. Also, few research studies were presented in the International Association for Dental Research meetings [40, 41]. Furthermore, searching the website of National Institutes of Health Trials indicted that nine randomized clinical trials utilizing SDF agents were registered in Egypt. In conclusion, SDF agents are available in Egypt. However, general dentists do not extensively use them. This may be due to either the lack of information about this treatment or concerns about the discoloration after SDF application. Consequently, the undergraduate dental curriculums in Egypt, particularly the cariology courses, should be updated.

## Finland

Finland and other Scandinavian countries (Denmark, Iceland, Norway, and Sweden) all share similar dental care systems. Dental care is usually subsidized and provided free for children and adolescents, with an emphasis on regular dental checkups and preventive dental care from the early ages. As a result, the proportion of caries-free children has increased substantially in the last decades, whereas the aging population retaining their own teeth incites new challenges for dental care [42, 43].

Even though the fluoride varnishes or other fluoride products are widely used in Scandinavian countries and are included in the national treatment recommendations, SDF use has not been very common for caries management until recently. SDF is commercially available in all Scandinavian countries, and the only product currently available is Riva Star (SDI Limited, Australia). Using Riva Star, a desensitizing agent, in the management and arresting of dental caries, is an off-label use, but the dental professionals can decide its use based on the available evidence and informed consent of patients. In Finland, SDF use was included in the recent caries management guidelines as a desensitizing agent and for eliminating biofilm, with the recommended use mainly focused on root caries patients [16]. Other Scandinavian countries also use it for elderly patients, or early childhood caries lesions, but its use is not included in national recommendations. There is no specific compensation for SDF use, but it is usually included in the prevention codes used with traditional fluoride varnish applications. Currently, there is no national/regional dental program, using SDF. Teaching on SDF use is generally included in the undergraduate and postgraduate education programs in Scandinavian countries, and its use in the clinics of the undergraduate programs varies among schools. Continuing education on SDF use is usually available for dentists through courses that dental organizations and manufacturers arrange, and there is an increasing research interest for its use for caries prevention.

## Ghana

SDF is not available in the Ghana market. There are no officially announced guidelines by the Ghana Dental Association or any other association or government bodies on SDF procurement and use. However, private practitioners can import SDF and use it in their own clinics, but the number of practitioners using it is very limited. The Ghana College of Physicians and Surgeons started a pediatric dentistry program in 2016. The Orthodontic and Pedodontic Department of the University of Ghana Dental School imported SDF from India for use in the school's pediatric clinic, aiming to generate research-related information on SDF. However, its use has been also limited in the university clinic because it has not been easy getting parents to understand and accept the black staining SDF causes. Information on SDF has been introduced in the postgraduate training but not the undergraduate training at the University of Ghana Dental School.

## India

SDF came to the Indian market in the past 7–10 years, and its use is limited. Four commercial brands of SDF are available, which are listed in Table 1. One of these (e-SDF) is an Indian product, while the rest are imported brands. The Drug Controller General of India, normally, does not restrict the usage of topically applied drugs, which are allowed by the US Food and Drug Administration (FDA), although specific permission has not been issued yet in India. At this moment, there are also no officially announced guidelines of SDF use by dental professional associations or research bodies. The Ministry of Health and Family Welfare of the Government of India is operating the National Oral Health Program under the flagship of the National Health Mission [44]. The key features of the program do not have specific recommendations for SDF application for caries arrest or prevention. Most of the health insurance schemes do not cover dental treatments (including SDF application) for reimbursement purposes. The training in SDF usage during undergraduate dental courses is rare. However, some of the institutes have included SDF in postgraduates training [45].

Recently, SDF use has been gaining popularity in India. There are a good number of Indian researchers trying SDF in dentistry for various purposes, such as using SDF as pulp capping material [46, 47] and as an endodontic medicament [48]; comparing the antibacterial and antiplaque effects of SDF with fluoride varnish and acidulated phosphate fluoride gel [49]; comparing the caries treatment effect of using SDF and ART in combination with the conventional drill and fill technique in primary molars; and investigating parental acceptance and perceptions of operators of SDF [50, 51].

## Japan

During the Edo period (1603–1868) in Japan, wealthy married women had a custom of “OHAGURO” (blacken teeth), which was a makeup method that dyed teeth black where tannins and ferrous acetate were used. In the 1960s, Professor Yamaga was inspired by “OHAGURO” because it covered and protected the tooth surface. With the aim of strengthening the tooth structure, he then developed the Diammine Silver fluoride [Ag(NH_3_)_2_F, molecular weight: 160.93] [52], and Prof. Nishino proved that SDF had a remarkable effect on suppressing the progression of ECC [53]. In 1970, TOYO Pharmaceutical Co., Ltd. (Osaka, Japan) productized a 38% aqueous solution of SDF, and BEE BRAND MEDICO DENTAL (Osaka, Japan) launched it as Saforide®. In the flood of ECC in Japanese children, until about 40 years ago, SDF relieved dental suffering of many children. After that, for a long time, the use of SDF had been avoided due to the aesthetic problem of black staining on carious lesions. However, in recent years, with the advent of a super-aging society, the frequent occurrence of root caries has become a serious problem in elderly people, requiring long-term care and for patients with xerostomia. Root caries is a problem for dentists because it, often, cannot be adequately restored. Under these circumstances, SDF, which has been around for half a century since its development, is once again in the limelight as a solution for root caries. Since the manufacturing patent of Saforide® has expired, SDF discovery is now active worldwide.

Japan first introduced its national health insurance in 1938. The Ministry of Health approved SDF in 1970 as a medical drug that helped to control the progression of dental caries. Approval indications are the suppression of early caries progression, secondary caries, and dentin hypersensitivity. SDF treatment costs $4.40 USD (460 Japanese Yen) for up to three teeth and $5.40 USD (560 Japanese Yen) for four or more teeth. Dental hygienists can use it under the direction of dentists. SDF is included in undergraduate curriculums, including dental materials, preventive dentistry, restorative dentistry, and cariology; it is also included in the national license examinations of dental and dental hygienist students. The Japanese Society of Conservative Dentistry recommended SDF to control the progression of root caries in the 2015 Clinical Practice Guideline for Caries Treatment [17]. In 2019, the Japanese Society of Gerodontology recommended that SDF was effective in suppressing the progression of root caries in patients with difficulties to access or receive dental care, such as bedridden patients and patients with dementia. However, the patients and their families should be well-informed that the carious lesions will turn black and the surrounding mucosa might become white transiently after SDF application.

## Kenya

There is an interim approval for using SDF in Kenya by the Pharmacy and Poisons Board of Kenya, which allows importing SDF products. There are no national guidelines for SDF use. Dental practitioners may use SDF according to other guidelines, such as those the International Pediatric Dental Associations issued. There is no official insurance that wholly covers dental treatment in Kenya, and most of the private insurances do not cover dental treatment either. The few insurance companies that offer dental plans will usually cover the cost for using SDF and charge as a fluoride application/therapy. Some patients have concerns about SDF because of the discoloration. However, since SDF treatment is inexpensive and non-invasive, most of the parents will usually accept the treatment to be applied on their children. SDF has not been incorporated into any official dental programs. There is no formal evidence generated on the use of SDF in the country. SDF is included in undergraduate and postgraduate dental training curriculums as a form of fluoride therapy.

## Mongolia

Although a number of national oral health programs have been implemented, the prevalence of dental caries remains high in Mongolia [54, 55]. In the ongoing governmental program Healthy Teeth—Healthy Child (2019–2023), SDF application for arresting cavitated carious lesions in primary teeth is recommended as part of the necessary dental services to be delivered. For the realization of the program, the Ministry of Health of Mongolia involved nationwide private dental clinics and allocated a separate budget to subsidize the dental treatments. It covers $21 USD for each child aged 2–6 years in the first year for oral examination, treatment (including SDF), and plans to increase the budget up to $46 USD from the second year until the end of the program for children aged 7–12 years old. As a result, between July 2019 and October 2020, a total of 176 private clinics have participated in the program, and SDF was applied on 55,761 deciduous teeth of 145,028 examined children [56].

In 2009, with the use of a translated version of the Japanese Pediatric Dentistry textbook, the topic of SDF use was further developed into a comprehensive curriculum for undergraduate training of Mongolian dentists nationwide. The Objective Structured Clinical Examination of National Dental Examination includes the SDF treatment procedure as a part of one examination station. The inclusion of an SDF syllabus in the dental curriculum enables students to be competent in using SDF to manage dental caries after their graduation. Because of the adoption of SDF in governmental programs and inclusion of SDF in the dental curriculum, SDF has become popular in Mongolia in recent years. The Japanese product Saforide (BEE BRAND MEDICO DENTAL, Japan) dominates the market, and dentists highly prefer it.

A standard guidance and recommendation for SDF management (MNS 5372-6) were composed in 2004 by the School of Dentistry of Mongolian National University of Medical Sciences, which play a key leading role in developing evidence-based policy documents in oral/dental care in Mongolia. It was generated for dental practitioners to implicate SDF in caries management in children under 12 years old and, therefore, to enhance a dental caries management outcome by arresting them. The guideline lists the definition and description of standard dental settings/facilities as well as the necessary instruments for SDF treatment. It also provides recommendations for a chairside guide with clinical application/reapplication steps, safety precautions, and reflections on possible side effects and complications.

## Nigeria

SDF is not officially available in the Nigerian market. There is also very little conversation in the public space about using SDF in managing caries. Only a few dentists have imported SDF products (such as Advantage Arrest) on their own and used them mainly on young children. SDF is not commonly included in the curriculum of undergraduate and postgraduate dental training in the country. Only one of the 11 dental schools reportedly holds seminars for undergraduate and postgraduates on using SDF in Nigeria. This same school has ongoing plans to conduct a postgraduate study on the use of SDF for managing ECC. In 2020, the National Oral Health policy for Nigeria started including a section on the use of SDF for managing ECC.

## South Africa

SDF, only recently, has become available in South Africa, and, to date, there are no official national policies or recommendations for using SDF as a caries-arresting agent. SDF is not readily available in most government clinics that serve the majority of the South African children. Some practitioners in private practice have started using it for indirect pulp therapy and for caries arrest with informed consent from the parents/legal guardians. There is no well-designed research studying the perception of the public of SDF. Some individual feedback from private practitioners suggested that SDF use was not popular due to the unaesthetic appearance. Currently, there are only two imported SDF products available in South Africa, namely Riva Star and e-SDF. SDF does not have a separate clinical code as a caries-arresting agent, and it is billed as a desensitizing agent or professionally applied topical fluoride. Students are exposed to the theory and clinical use of SDF in both undergraduate and postgraduate training programs. SDF, also, is being used in tertiary institutions for pediatric patients and patients with special health care needs. The Paedodontic Society of South Africa (PSSA) has recognized the clinical benefits, cost-efficiency, and ease of clinical use of SDF. Therefore, the PSSA would like to promote the SDF use in South Africa, especially targeting children living in rural and underprivileged areas. The PSSA has an educational campaign planned to educate the public and dental practitioners on SDF use. It will also initiate communications with the government to have SDF be widely available in all dental clinics in South Africa.

## Switzerland

Although an impressive decline in dental caries has been observed in most industrialized countries in Europe over the past four decades in several age groups, caries still remains a major childhood health problem, especially in young children, which is a peculiar pattern of caries in toddlers aged 12–30 months [57]. This has led to an interest in using products, such as SDF for target populations. However, because SDF is labeled with the indication for treating dentin hypersensitivity in Switzerland and most European countries, and the off-label use for caries management on children is strict, the general use of SDF in Switzerland and across Europe (France, Germany, Greece, Ireland, Italy, Netherlands, Portugal, and Spain) is very limited. So far, there is only one SDF product, Riva Star [58], commercially available in the aforementioned European countries. There are no formal national programs that use it. Dental schools in Switzerland and most European countries are teaching SDF didactically. However, SDF use in the clinics of the dental schools is limited mainly on children who have special needs or who cannot be cooperative with conventional treatments. In addition, in the countries where the SDF is only available in unit dose capsules (such as Greece and France), high expense is also mentioned as a deterrent for its use.

Although most national dental societies have restrained from publishing SDF-specific protocols or guidelines, recently, clinical guidance and recommendation for caries management have been jointly published by the European Academy of Pediatric Dentistry, the Organization for Caries Research and the European Federation of Conservative Dentistry. This advice, using fluoride, in particular SDF (a high strength of recommendation and consensus), can be used to treat dentinal carious lesions without pulpal involvement in young children [18–21].

## Thailand

Thailand had initially imported SDF from Japan (Saforide) for research purposes and caries control in some remote areas with high prevalence of ECC. Studies of effectiveness on arresting dentinal carious lesions, optimum frequency of application, and the cost-effectiveness of SDF in the Thai population since 2009 have confirmed the positive results reported in other countries [59–61]. In 2017, the Dental Association of Thailand launched a guideline for using fluoride, including SDF, suggesting SDF application semiannually on dentinal carious lesions until caries arrest for patients with severe ECC, uncooperative behaviors, or compromised health conditions; followed by another guideline on caries diagnosis and management released in 2018, which recommended SDF as an alternative treatment of dental caries (ICDAS 5 and 6) in primary teeth when access to dental care was limited as well as root caries in permanent teeth [22]. There upon, in late 2018, the FDA Thailand cleared the first and only commercial product of 38% SDF (Topamine^TM^, DentaLife, Australia) as a class II medical device for professional tooth desensitization.

Knowledge of SDF use off-label for arresting caries is incorporated into the dental school curriculum in Thailand, including both undergraduate and postgraduate trainings related to children and special and geriatric patients. Some dental schools introduce SDF use in school programs. However, clinical experience of students of SDF varies. Dental schools and organizations host continuing education on SDF use, which is periodically available for dentists. Although there is no established national/regional healthcare program using SDF, the Ministry of Public Health Thailand has made SDF available in dental services for managing coronal caries in primary teeth and root caries in permanent teeth. The SDF application is categorized as a type of professionally applied fluoride covered by Universal Health Coverage at no cost.

## The United Kingdom

SDF is commercially available in the United Kingdom (UK), and the only product widely available is Riva Star. Similar to other products, such as fluoride varnish, with the exception of Duraphat, Riva Star remains an off-label product for managing and arresting dental caries. Thus, in 2020, Riva Star, which is Conformitè Europëenne, was marketed for use as a desensitizing agent, even though it is not licensed for arresting caries, but can be used “off label”, by a registered dental professional when the prescriber judges it to be in the best interests of the patient, on the basis of the available evidence [62]. The majority of children in the UK have dental care under the National Health Service (NHS), and, in each of the four countries, the NHS remuneration to dentists varies. However, no devolved administration has yet agreed on a specific payment for SDF, which is over and above a traditional fluoride varnish application. This makes using SDF under the NHS payment structure uneconomical for most dentists. Presently, there is no national/regional dental program using SDF, and its widespread use will be determined by the NHS changing its renumeration fee for its use. Teaching on SDF use is now generally provided in UK undergraduate dental schools and in postgraduate dental programs [58].

The recent outbreak of SARS-CoV-2, responsible for the coronavirus disease (COVID-19), has brought a new focus on the working environment of dentists in clinical settings. The risk to dental providers, their staff, and patients to airborne particles produced by high-speed rotary instruments has led to a number of mitigation procedures. In 2020, the Department of Health (England) highlighted using SDF as a non-aerosol procedure, which could be used to help patients, especially with regard to urgent dental care situations [23]. At the same time, the British Society of Pediatric Dentistry published a resource pack on SDF and, in particular, its use in the COVID pandemic phase [24].

## The United States

SDF was approved in the US by the FDA in 2014 at a 38% concentration as a device to treat dentinal hypersensitivity in patients over 21 years of age. The only manufacturer is Elevate Oral Care, which markets the product as Advantage Arrest in a bottle containing 8 ml of blue-tinted liquid and as unit-dose ampules with 0.1 ml each. An average drop measures 33 microliters and contains approximately 1.7 mg of fluoride and 8.5 mg of silver [63]. Because of its proven efficacy to arrest dentinal caries in children, and considering the burden of disease in the US population [64], the American Academy of Pediatric Dentistry (AAPD) published a guideline for its use in 2017 [25], with the intention of supporting its off-label use for the arrest of cavitated caries lesions in primary teeth as part of a comprehensive caries management program (conditional recommendation based on low-quality evidence). AAPD also published a policy [27] to support the following: its use with informed consent and within the goals of a dental home, third-party reimbursement, delegation of applying SDF to auxiliary dental personnel or other trained health professionals, education to ensure a good understanding of appropriate use, coding and billing practices, and further research on the topic. In 2018, the American Dental Association published recommendations to prioritize the use of SDF 38% over fluoride varnish to arrest advanced carious lesions in primary teeth (strong recommendation based on moderate certainty of evidence) and permanent teeth (conditional recommendation based on low certainty of evidence) [26]. Specific billing codes for caries arrest were developed subsequently; however, reimbursement from third-party payers remains heterogeneous, with great variation in coverage from publically funded insurances from state to state, and little or no buyout from private insurance companies. Government agencies funded several presently ongoing research projects with the aim of changing the labeling to the specific indication of caries arrest in children and to incorporate its use into prevention programs in schools in areas where children have a high burden of disease and poor access to or utilization of services. In addition to reimbursement challenges, acceptance of the black staining from parents and patients is often cited as a barrier to implementation. A recent study [65] has reported that parental acceptance is related to the visibility of the staining (anterior vs. posterior location), and that parents are often willing to overlook aesthetics to avoid more risky scenarios, such as sedation or general anesthesia. However, some parents are reluctant to accept the aesthetic challenge, and acceptance is mediated by ethnicity, education, and socioeconomic status, thus the importance of appropriate informed consent. In terms of training in academic environments, in 2015, 25% of postgraduate pediatric dental programs were using SDF in their clinics, while, in 2020, its didactic teaching and use are universal [66]. This trend is expected to translate into undergraduate education in the near future.

## Discussion

This is the first publication that summarizes the global policies, guidelines, and information of using SDF for caries management. It provides an overview of SDF use in different countries and allows dental professionals to obtain a general idea about SDF use worldwide. A convenient sampling method was used to recruit the countries in each continent. We searched SDF-related research articles in the literature and identified several researchers from different countries. We asked for their contribution to this paper; all of them agreed to join and shared the situation of SDF use in their own country. Apart from identifying researchers from the literature, snow-ball sampling was adopted as well. The contributors tried to suggest more experts in SDF research or clinical work in other countries from their academic networks. Finally, we collected reports from 17 countries. The choosing process is considered the major limitation of this study. It is not applicable to report the information of SDF use in all the countries around the world. It is also not appropriate to choose the representative countries to form this article. Nevertheless, some countries share the similar situation of SDF use, for example, the Scandinavia countries. The readers who are interested in other Scandinavia countries can refer to the Finland section.

We found that the SDF use situation varies significantly in different countries. Except for mainland China, SDF is currently available in most countries. The US and most European countries have developed their own guidelines for SDF use; it is often included as one section of clinical recommendations of caries management. There is no unified guideline of using SDF for caries treatment issued by any international associations or organizations. The reason may be that, although SDF has been proved to be effective in arresting dental caries, there is still no agreement between clinicians regarding the SDF treatment protocol. More research is needed to provide more evidence that can support an identical protocol of SDF therapy. Almost all dental schools include SDF in their dental training programs, mostly in pediatric dentistry sessions. However, the training sessions are usually brief, and specific clinical training is rare to find. This might be why some contributors have reported that the dentists in their countries did not know SDF well or use it frequently in their clinical work.

SDF has been accepted as a simple, non-invasive, and inexpensive treatment for dental caries in young children, elderly people, and people with special needs. It can also be a cost-effective strategy adopted in community-based programs. Recently, there have been two ongoing region-wide oral health programs, aiming at improving oral health of children, including SDF treatment as one of the components (one in Hong Kong and one in Mongolia). The results and evaluations of those programs may provide insights for researchers in other countries and promote SDF use in large-scale community programs or even national healthcare policies.

On the other hand, due to COVID-19 global pandemic, the World Health Organization has recommended that the dental clinics should minimize aerosol-generating procedures [67]. In general, traditional dental caries management involved using instruments, such as high-speed and slow-speed handpieces and the air-water syringes, that might generate aerosols, containing tooth debris, saliva, and blood. However, SDF is considered a non-aerosol-generating treatment because there is no need for carious tissue to be removed [68]. Clinical practitioners always simply apply SDF by a micro brush on the carious lesion after drying the lesion with cotton. Therefore, to reduce the aerosols generation and the risk of transmission of bacteria and viruses in dental settings, SDF can be considered an alternative strategy for caries management under the COVID-19 outbreak.

## Conclusion

SDF is available in most countries in the world. Different countries have different policies and guidelines for SDF use in managing dental caries. SDF products are readily available worldwide; some countries, including Argentina, Australia, Brazil, India, Japan, Thailand and the US, have their own SDF products. SDF is accepted as a promising and non-invasive strategy to treat dental caries and has been adopted in different community-based dental programs.

## Author Contributions

CC and EL contributed to conception and design of the study. SG, GA, PA, KB, RB, GC, KC, AC, TC, YC, DD, MLF, MOF, AG, HH, VJ, AK, SL, PL, VM, TM, YM, NP, and AT-M wrote sections of the manuscript. SG wrote the first draft of the manuscript. All authors contributed to manuscript revision, read, and approved the submitted version.

## Conflict of Interest

The authors declare that the research was conducted in the absence of any commercial or financial relationships that could be construed as a potential conflict of interest.

## Publisher's Note

All claims expressed in this article are solely those of the authors and do not necessarily represent those of their affiliated organizations, or those of the publisher, the editors and the reviewers. Any product that may be evaluated in this article, or claim that may be made by its manufacturer, is not guaranteed or endorsed by the publisher.
